# Comprehensive Evaluation of the Safety and Efficacy of BAFASAL^®^ Bacteriophage Preparation for the Reduction of *Salmonella* in the Food Chain

**DOI:** 10.3390/v12070742

**Published:** 2020-07-10

**Authors:** Ewelina A. Wójcik, Małgorzata Stańczyk, Arkadiusz Wojtasik, Justyna D. Kowalska, Magdalena Nowakowska, Magdalena Łukasiak, Milena Bartnicka, Joanna Kazimierczak, Jarosław Dastych

**Affiliations:** Proteon Pharmaceuticals S.A., Tylna 3a, 90-364 Łódź, Poland; mstanczyk@proteonpharma.com (M.S.); awojtasik@proteonpharma.com (A.W.); jkowalska@proteonpharma.com (J.D.K.); mbyknowakowska@gmail.com (M.N.); mlukasiak@proteonpharma.com (M.Ł.); mbartnicka@proteonpharma.com (M.B.); jkazimierczak@proteonpharma.com (J.K.); jdastych@proteonpharma.com (J.D.)

**Keywords:** bacteriophages, safety, *Salmonella*, antibiotic alternative

## Abstract

Bacteriophages are bacterial predators, which are garnering much interest nowadays vis-à-vis the global phenomenon of antimicrobial resistance. Bacteriophage preparations seem to be an alternative to antibiotics, which can be used at all levels of the food production chain. Their safety and efficacy, however, are of public concern. In this study, a detailed evaluation of BAFASAL^®^ preparation was performed. BAFASAL^®^ is a bacteriophage cocktail that reduces *Salmonella* in poultry farming. In vivo acute and sub-chronic toxicity studies on rats and tolerance study on targeted animals (chicken broiler) conducted according to GLP and OECD guidelines did not reveal any signs of toxicity, which could be associated with BAFASAL^®^ administration. In addition, no evidences of genotoxicity were observed. The tolerance study with 100-times concentrated dose also did not show any statistically significant differences in the assessed parameters. The in vitro crop assay, mimicking normal feed storage and feed application conditions showed that BAFASAL^®^ reduced the number of *Salmonella* bacteria in experimentally contaminated feed. Moreover, reductions were observed for all examined forms (liquid, powder, spray). Furthermore, the in vivo efficacy study showed that treatment with BAFASAL^®^ significantly decreased *Salmonella* content in caeca of birds infected with *Salmonella* Enteritidis. Detailed examination of BAFASAL^®^ in terms of safety and efficacy, adds to the body of evidence that bacteriophages are harmless to animals and effective in the struggle against bacteria.

## 1. Introduction

Bacteriophages, also called phages, are viruses that play an essential role in regulating bacterial populations by triggering bacterial lysis [1]. Although phages were discovered over 100 years ago, a growing interest in their therapeutic potential has been observed over the last decade. Recently, phages have been implemented as antibacterial agents in the food industry to increase food safety [2], as well as in food animal production and crop protection to prevent bacterial animal and plant diseases, respectively [3,4].

There are a number of arguments supporting the claim that bacteriophages are inherently safe for humans and animals. Bacteriophages are the most common biological entities in every terrestrial and aquatic habitat on Earth. Their number is estimated at 10^31^, and is ten times higher than the number of bacterial cells [5,6]. In seawater, the phages occur at densities of up to 2.5 × 10^8^ particles per milliliter [7]. Therefore, our bodies are frequently and continuously exposed to high numbers and various collections of phages [8]. Bacteriophages are also the most diverse microbes present in human microbiota. Numerous analyses have confirmed the abundance of phages in respiratory tract, vaginal, skin, oral, or intestinal microbiomes [9,10,11,12,13,14]. In human feces, there is at least 10^12^ phage particles per gram of sample. Such persistent non-pathogenic association between phages and higher organisms seems possible, as viral replication occurs only in bacterial hosts, which are also stable members of the microbiota [15]. Phages cannot infect eukaryotic cells because the structure of the cell surface receptors and intracellular organization differ significantly from their bacterial hosts. Phages, however, are capable of freely spreading throughout the body of higher vertebrates. They have been detected within regions of the body, which are considered sterile, including the blood, lymphs, and internal organs [16]. It should also be noted that bacteriophages are constantly consumed, as high numbers of bacteriophage particles are components of the normal microflora of a wide variety of raw (e.g., fresh meat and oysters) and fermented food products (e.g., commercial sauerkraut, yoghurt and cheese) [17,18,19,20,21,22].

Additional evidence supporting the safety of bacteriophages is provided by oral toxicity studies. Carlton et al. and Kang et al. showed that exposure of experimental animals to high bacteriophage doses (2 × 10^12^ PFU/kg body weight and 1 × 10^11^ PFU/kg body weight, respectively) had no effect on animal deaths and clinical signs in comparison to the control group, indicating the non-toxic effect of bacteriophage administration. Furthermore, no abnormal behavior or changes in physical appearance in experimental animal models were observed [23,24]. The safety of bacteriophages was also evaluated in mice treated with two concentrations (2 × 10^7^ PFU/10 g body weight, and 2 × 10^6^ PFU/10 g body weight) of anti-*E. coli* bacteriophage cocktails. The results of this work demonstrate that bacteriophages do not induce any toxic effect in experimental animals [25]. The absence of evidence of toxicity of bacteriophages was also observed in an acute toxicity assay performed by Chen et al., in which BALB/c mice inoculated intraperitoneally with PHB02 phage at a dose of 1 × 10^8^ PFU/animal did not reveal any behavioral changes [26].

The safe and successful application of bacteriophages for therapeutic purposes has also been proven in human clinical trials. Bruttin et al. performed a safety study on healthy volunteers and observed that the therapy was well-tolerated. Mild adverse effects were observed and assessed as unrelated to the study intervention. Moreover, the blood biochemical parameters of the study subjects had normal values [27]. Similar results and conclusions were drawn by Sarker et al. [28]. They did not observe any adverse effects of phage therapy. Moreover, Rhoads et al. did not show any adverse events in phase I of their safety clinical trial of a phage cocktail approved by the FDA (WPP-201) [29]. Consistent results were also demonstrated by Wright et al. [30], and Hoyle et al. [31]. In 2018, Gindin et al. published the results from safety and tolerability clinical studies. In this study, participants treated with four bacteriophage strains included in the PreforPro commercial preparation did not show any differences in the studied parameters compared to the untreated control group. The authors concluded that consumption of therapeutic doses of cocktail of four bacteriophages was both safe and tolerable for the target healthy, human population [32].

A number of studies indicates that phages may be a useful tool for the control of foodborne pathogens [33]. In recent years, several bacteriophage-based products targeting *Salmonella* spp., *Listeria monocytogenes*, *Shigella* spp., and *Escherichia coli* (*E. coli*) O157:H7 were approved as Generally Recognized as Safe (GRAS) by the U.S. Food and Drug Administration (FDA), and used for pathogen control in food processing, including ready-to-eat foods and poultry products [34,35].The results of several studies have highlighted the successful application of commercially available phage-based products in food [36,37,38,39]. 

In the current study, the safety and effectiveness of anti-*Salmonella* bacteriophage cocktail BAFASAL^®^, intended to prevent contamination of dried and liquid (electrolyte solution) poultry feed was investigated. In a series of experiments, toxicity, genotoxicity, tolerance, and *Salmonella* numbers following artificial contamination of feed were assessed.

## 2. Materials and Methods

### 2.1. BAFASAL^®^ Preparation

BAFASAL^®^ is a cocktail of four anti-*Salmonella* bacteriophages, which were deposited in the Polish Collection of Microorganisms under accession numbers: PCM F/00069 (strain 8 sent 1748), PCM F/00070 (strain 8 sent 65), PCM F/00071 (strain 3 sent 1), and PCM F/0097 (strain 5 sent 1). The high titer bacteriophage lysates were subjected to genomic DNA extraction using the modified method of Su et al. [40]. Briefly, bacteriophage lysates obtained after propagation on the host strain were subjected to DNase I for disrupting debris of bacterial DNA. Then, for pelleting the phage particles, 2M ZnCl_2_ solution in 1:50 (*v*:*v*) was used. Next, the phage pellet was dissolved in a TENS buffer (50mM Tris-HCl, pH 8.0, 100 mM EDTA, 100 mM NaCl, 0.3% SDS) supplemented with proteinase K, which disrupted phage capsids. Deproteinated phage DNA was subjected to extraction by a solution of phenol/chloroform/isoamyl alcohol (25:24:1). Eluted DNA concentrations were measured using a BioSpectrometer^®^ (Eppendorf, Hamburg, Germany) and stored at −20 °C for RFLP analysis. In RFLP analysis, the digestion reaction was performed by incubating 1 μg of isolated DNA with 2.5 U of *Eco*RI enzyme (ThermoScientific, Waltham, Massachusetts, United States) in a final reaction volume of 20 μL at 37 °C for 2 h. The restriction fragments were separated by 1.5% agarose gel electrophoresis in a TAE buffer for 2 h at 30 V and stained by the nucleic acid stain (SimplySafe™, Eurx, Gdansk, Poland).

Then, a TEM micrograph of each phage was taken in the Laboratory of Microscopic Imaging and Specialized Biological Techniques of the Faculty of Biology and Environmental Protection, University of Lodz, using a JEM 1010 electron microscope (JOEL Ltd., Tokyo, Japan) at 80 kV according to the method described by Maszewska et al. [41]. Bacteriophage lysates were centrifuged at 24,500 × *g* for 3 h at 4 °C. Then, the phages were washed twice with 5% ammonium molybdate solution (Sigma-Aldrich, St. Louis, Missouri, United States) and pH 6.0, using the above spin conditions. The final sediments were suspended in 5% ammonium molybdate to obtain the titer of 1 × 10^11^ PFU/mL (PFU—plaque forming unit). Subsequently, one drop of the phage suspension was placed onto the formvar and carbon-coated 200-mesh copper grid (Polysciences Inc., Warrington, USA) and drained for 3 min. Then, samples were negatively stained for 45 s with 2% (*w*/*v*) phosphotungstic acid (PTA) in darkness. 

Finally, bacteriophage genomes were sequenced by whole-genome shotgun sequencing: 3sent1, 8sent1748 and 5sent1 on the Roche 454 and 8sent65 on the Illumina platform. The draft genomes were de novo assembled by CLC Genomic Workbench. Next, bioinformatic analysis were performed starting with annotation of assembled genomes which was carried out automatically using DNA Master v 5.23.2 based on GeneMarks and Glimmer algorithms (tool written by Dr. Jeffrey Lawrence, the University of Pittsburgh) with necessary manual corrections. Then, reference sequences were found using the Basic Local Alignment Search Tool (BLASTn), NCBI which allowed to classify analyzed bacteriophages into taxonomic groups. Determination of lytic or lysogenic lifecycle was performed on the basis of careful analysis of each ORF (open reading frame) determined by DNA Master. It was performed in BLASTp, InterPro (https://www.ebi.ac.uk/interpro/, accessed on 3 June 2020)—a tool to predict protein domains - and in HHPred at web service MPI Bioinformatics Toolkit (toolkit.tuebingen.mpg.de/#/tools/hhpred; accessed on 3 June 2020) which finds remote homologs of query amino acid sequences. Finally, a PhageAI tool was used to confirm manual predictions—a platform to determine lifecycle of phages with the help of Machine Learning and Natural Language Processing (https://phage.ai/, accessed on 19 June 2020). Identification of antibiotic resistance genes and virulence factors coding for toxins, pathogenic islands or adhesins was performed with the help of online tools from CGE server: ResFinder 3.2 [42] and VirulenceFinder 2.0 [43] as well as on the basis of ORF analysis. The genome sequences of phages described in this study were deposited in GenBank under accession numbers: MT653143-MT653146.

### 2.2. BAFASAL^®^ Production

Each bacteriophage included in BAFASAL^®^ was amplified in a separate culture. In this procedure, the culture medium was inoculated with the host production strain and incubated at 37 °C for approximately 2 h. Next, the selected phage was added to the bacterial culture, and incubation proceeded for a further 3 h at 37 °C leading to cell lysis. This process enables to obtain a high titer of amplified bacteriophage at a level of about 1 × 10^9^ PFU/mL. Upon amplification, the biomass (unlysed cells and cellular debris) was separated from the phage-containing culture fluid by filtration. Once the amplification procedure was completed, the titer of the bacteriophage was assessed by phage enumeration using a double agar overlay plaque assay and calculated for a mixture of four different bacteriophage suspensions at equal titer value. Finally, the preparation was completed with sterile water to obtain BAFASAL^®^ at the titer of 1 × 10^8^ PFU/mL. The final product was monitored for endotoxins level. For preparing the dry form of the preparation, liquid BAFASAL^®^ was mixed with trehalose and spray-dried yielding a powder at the titer of 1 × 10^7^ PFU/g. Both forms of BAFASAL^®^ were finally tested by phage enumeration using the double agar overlay plaque assay, analyzing the microbiological sterility of the product and the presence of bacterial genomic residues according to EFSA’s “Guidance on the characterization of microorganisms used as feed additives or as production organisms.” [44].

### 2.3. BAFASAL^®^ Host Range

The host range was determined via spot test on 313 *Salmonella* strains isolated from seek animals on poultry farms. Bacterial lawns of each strain were made in triplicates using the double agar overlay method, on which 20 µL droplets of the BAFASAL® preparation (at the titer of 1 × 10^8^ PFU/mL) were applied. After overnight incubation, the degree of lysis of the lawns was determined. The spot test was repeated three times for each strain. The following spot evaluation system was used: sensitive strain: completely clear spot or turbid spot, resistant strain: no clearing.

### 2.4. Animal Safety Studies

#### 2.4.1. Acute Oral Toxicity Study

The acute oral toxicity study was performed in the Department of Toxicological Studies at the Institute of Industrial Organic Chemistry, Branch Pszczyna, with the approval of the Local Ethical Committee for Animal Experimentation in Katowice (Resolution No. 108/2015 of 31 July 2015). The study was conducted in compliance with the Good Laboratory Practice principles and in accordance with the OECD guidelines (OECD Environment, Health and Safety Publications).

The experiment was conducted on female Wistar rats (Cmdb: WI; outbred) obtained from the husbandry of laboratory animals of the Experimental Medicine Center at the Medical University in Białystok. In this experiment, 11-week-old females with an average body weight ranging from 202.3 g to 214.0 g were included. The animals were kept in air-conditioned rooms and were housed in plastic cages covered with wire bar lids. There were three animals in each cage. Prior to BAFASAL^®^ administration, the animals were given food and water ad libitum. The study was performed in 4 stages. At each stage, 3 female rats were subjected to a particular single BAFASAL^®^ dose, i.e., stages 1 and 2—dose of 300 mg/kg of body weight (b.w.); stages 3 and 4—dose of 2000 mg/kg b.w. The bacteriophage preparation in the form of an aqueous solution at a volume of 0.5 mL/100 g b.w. was administered through a metal stomach tube. Prior to administration, the animals had been fasted for about 19 h. After the administration of BAFASAL^®^, the animals were observed for 14 days. General and detailed clinical observations were conducted daily. Clinical features, including alterations in locomotor system, behavior, reactions to stimuli, skin and hair, eyes and eyelids, and the respiratory, digestive and reproductive systems were assessed. The evaluation of the general condition of the animals, i.e., the observation of all animals for morbidity and mortality, was conducted once or twice a day during the 14-day experiment. The body weights of the animals were determined directly prior to administration, and at days 7 and 14. After the 14-day observation period, the animals were euthanized and subjected to a detailed gross examination. This involved the observation of the external body surface, all natural apertures, and the cranial, thoracic, and abdominal cavities and their contents.

#### 2.4.2. Sub-chronic Oral Toxicity Study

A sub-chronic oral toxicity study using repeated dosage (for 90-days) in rodents was performed by the SORBOLAB Research Laboratory, Poznań, with the approval of the Local Ethical Committee for Animal Testing in Poznań (Resolution No. 43/2017). The study was conducted in compliance with GLP and in accordance with the OECD guidelines (OECD Environment, Health and Safety Publications). One hundred Wistar rats, fifty young males and fifty young females (nulliparous and non-pregnant), 6 weeks old, were used in the study. Animals were provided by the Poznan University of Medical Sciences, Department of Toxicology. The animals used in the research underwent an 8-day quarantine and 14-day acclimatization. During the study, the animals were housed in rooms with the following parameters: temperature mean ± SD: quarantine 20.1 ± 1.4 °C, acclimatization and study 21.8 ± 1.0 °C; humidity mean ± SD: quarantine 54.0 ± 6.6%, acclimatization and study 57.0 ± 7.9%. 

Animals were divided into six groups: three groups receiving three different doses of bacteriophage preparation, a control group, a satellite group and a control satellite group. The doses of test materials were: 2000 mg/kg b.w., 500 mg/kg b.w., and 125 mg/kg b.w. BAFASAL^®^ was orally administered as a water solution using a stomach tube, once a day, in the same volume (1.5 mL) for 90 days, seven days a week. The control group (10 females and 10 males) received vehicle (ultraclean water from Millipore Synergy UV system by Merck), in the same volume as the test material. The animals from the satellite group (5 females and 5 males) received test material in a dose of 2000 mg/kg b.w., and the control satellite group (5 females and 5 males) received ultraclean water from Millipore Synergy UV system by Merck. The test material was administered in the same volume (1.5 mL/animal/day). During the length of the study, all animals were observed closely for signs of toxicity. After the administration period, the animals from the study groups and the control group were euthanized, and tissues and organs underwent histopathological examination. The satellite and control satellite groups were observed for the following 14 days in order to detect any delayed development of potential toxic effects. During the dosing period, detailed clinical observations were performed once a week in order to detect signs of toxicity. Attention was paid to alterations of skin, fur, eyes, mucous membranes, occurrence of secretions and excretions, vegetative activation (e.g., lacrimation, piloerection, pupil size, respiratory pattern), gait, posture, response to handling, and auditory stimuli, as well as the presence of involuntary movements or unusual behavior. All animals were weighted once a week. Measurements of food and water consumption were also made once a week. During the final week of the study, 24 h urine was collected from the animals and a general urine analysis was performed. At the end of the experiment, biochemical blood testing was performed, as well as histopathological analysis of internal organs. 

#### 2.4.3. Tolerance Study

The tolerance of BAFASAL^®^ was performed on broilers at the Department of Poultry Science, Olsztyn University of Warmia and Mazury. The study was conducted in compliance with the Good Laboratory Practice principles and in accordance with the EFSA guidelines. In this study, 1280 male and female one-day-old broilers (Ross 308) were used, which were purchased from a local commercial hatchery. The IB-Primer vaccine against infectious bronchitis was administered at the hatchery. Birds were allocated to four treatment groups so that each treatment was applied to 16 single-sex pens (8 male and 8 female pens) of 20 broilers. The study lasted 38 days. The four groups of birds were: T1—control group, T2—group receiving BAFASAL^®^ at 2 × 10^6^ PFU/bird/day, T3—group receiving BAFASAL^®^ at 2 × 10^7^ PFU/bird/day (dose 10×), and T4—group receiving BAFASAL^®^ at 2 × 10^8^ PFU/bird/day (dose 100×). Broilers had free access to mash diets, and water was provided *ad libitum*. Commercial diets were used, which were nutritionally balanced and met or exceeded the nutritive requirements of the birds for growth. During this experiment, the following parameters were recorded: pen body weight at 0, 12, 22 and 35 days of trial; average daily weight gain; average daily feed intake and feed; gain calculated from 0–12, 13–22, 23–35 and 0–35 days on trial; pen water intake measured at weekly intervals. Moreover, blood samples were collected on days 35–38 of the trial for routine hematology and biochemistry analyses. Finally, daily health records, illness, culls, mortality, including reason for culls and probable cause of mortality, were recorded.

### 2.5. Genotoxicity Studies Including Mutagenicity

Two separate in vitro tests were carried out to evaluate the genotoxicity potential of BAFASAL^®^. Both studies were conducted at Selvita S.A. (Krakow, Poland). The evaluation of the genotoxic and mutagenic potential was performed for 50× concentrated BAFASAL^®^. The genotoxic potential was assessed with a micronucleus test (MNA), and the mutagenic potential was evaluated with the Mouse Lymphoma Assay (MLA). Both genotoxicity tests were preceded by preliminary cytotoxicity tests, which allowed to determine the non-toxic range of BAFASAL^®^ concentrations. In the MNA assay, CHO-K1 cells were exposed to different concentrations of BAFASAL^®^ and negative and positive control compounds (cyclophosphamide (Cp) and mitomycin C (MMC)) either with (+S9) or without (−S9 short and extended treatment) an exogenous metabolic activation. The determination of the non-cytotoxic range of BAFASAL^®^ concentrations in the MLA assay was tested in the presence and absence of S9 exogenous metabolic activation with 4 h exposure, and in the absence of metabolic activation with 24 h exposure.

#### 2.5.1. Genotoxicity Test (MNA)

The analysis of genotoxic potential was performed in accordance with guideline 487 of the Organization for Economic Cooperation and Development (OECD) and under GLP requirements. The assay was performed on a CHO-K1 cell line. The cells were exposed to five concentrations of BAFASAL^®^ (0.25 µL/mL, 0.5 µL/mL, 1 µL/mL, 2 µL/mL and 5 µL/mL) both with (+S9 short treatment) and without (−S9 short and extended treatment) exogenous metabolic activation. Cells were cultured in Ham’s F12 medium supplemented with 10% of FBS (Fetal Bovine Serum) and antibiotics (100 U/mL penicillin and 100 µg/mL streptomycin). The cells were cultivated at 37 °C in a humidified atmosphere containing 5% CO_2_. The procedure was as follows. For −S9 short and extended treatment experiments, cells were plated at 50,000 cells/well into a 24-well plate, in a total volume of 500 µL/well. For +S9 short treatment, cells were plated at 100,000 cells/well into 12-well plate, in a volume of 1000 µL/well. The cells were cultured overnight prior to the start of the assay. After 18–22 h incubation, the medium was removed and replaced with 500 µL or 600 µL (for −S9 and +S9 treatment, respectively) per well of medium with BAFASAL^®^ and appropriate positive and negative controls. For the short treatment (+/− S9), cells were treated with BAFASAL^®^ for 3 h, then the medium was removed, cells were washed once with a warm medium, and fresh medium containing cytochalasin B (3 µg/mL) was added for 25 h. For the extended exposure period (27 h), cells were stimulated with BAFASAL^®^ diluted in growth medium containing cytochalasin B (3 µg/mL). At the end of the incubation time, the medium was removed and cells were washed once with warm PBS. Then, they were detached by trypsinization, collected to 15 mL conical tubes and centrifuged. After additional centrifugation with PBS, cells were treated with 1 mL of warm KCl hypotonic solution for 60 s and were then fixed by adding 2 mL of cold fixative (acetic acid: methanol in proportions 1:3 *v*/*v*). The centrifugation and fixation steps were repeated once again. Then, the cells were incubated overnight in a fixation solution, at room temperature, followed by another centrifugation. Then, the supernatant was discarded, the cell suspension was gently re-suspended, and a few drops of suspension were placed on a cold clean glass slide in a humid chamber (45 °C in water bath) and air dried. The next day, the slides were stained with 15% Giemsa stain for 5 min, and washed twice with distilled water, air dried and analyzed.

#### 2.5.2. Mutagenicity Assay

The Mouse Lymphoma Assay (MLA) is a short-term assay designed to detect forward gene mutations induced by mutagens at the heterozygous thymidine kinase (TK) locus. It is capable of quantifying genetic alterations. For this study, it was performed on the recommended cell line L5178Y TK+/−. The performed procedure was strictly according to the OECD 490 guideline. The L5178Y TK+/− (clone 3.7.2C) cell line was purchased from American Culture Collection (ATCC). The cells were cultured in RMPI 1640 medium, supplemented with 10% (*v*/*v*) of heat inactivated serum. The concentration of heat inactivated horse serum was reduced to 5% (*v*/*v*) prior to the treatment of BAFASAL^®^. The mutagenic potential of 50× concentrated BAFASAL^®^ was assessed during a 4 h incubation with and without metabolic activation, and a 24 h incubation without metabolic activation. All incubations during the procedure were performed in the following conditions: 5% CO_2_, 37 °C. The definitive mutagenicity assay included the following samples: positive control (methylmetanosulfate—MMS in concentrations of 15 and 7.5 µg/mL in the absence of S9, and cyclophosphamide CP in concentrations of 3 and 1.5 µg/mL in the presence of S9), vehicle control to positive samples (PBS), vehicle control to BAFASAL^®^ (placebo) and four concentrations of BAFASAL^®^ (0.5 µL/mL, 1.0 µL/mL, 1.5 µL/mL, 2 µL/mL). Upon exposure to the test item, cell suspension from each culture was used for counting (post-treatment) and for plating immediately after treatment to obtain the Cloning Efficiency (CE) and Relative Total Growth (RTG) values. A portion from the cell suspension was used to prepare a three-step dilution with non-selective (without TFT) medium, to obtain 8 cells/mL concentration. The 200 µL of cell suspension were then dispensed to each well of two 96-well flat-bottom plates for each tested dose and control. Approximately 20–24 h after treatment, the cultures were counted and diluted with fresh medium to 2 × 10^5^ cells/mL and placed back for incubation. On the following day, the Relative Suspension Growth was established for each concentration of BAFASAL^®^ preparation. From observations on the recovery and growth of the cultures during the expression period, appropriate test dose levels demonstrating up to 90% suspension growth inhibition plus negative and positive controls were selected to be plated for Cloning Efficiency/Viability and Trifluorothymidine (TFT) resistance. The cultures were adjusted to a concentration of approximately 2 × 10^5^ cells/mL in cloning medium (containing 20% of serum), and incubated for 30 min in standard conditions. In the next step, the cells were diluted to the appropriate concentration to be plated for TFT resistance (2000 cells/well) and make a three-step dilution for cell viability plating. For the selection of TFT-resistant phenotype, the cell concentration was adjusted to 1 × 10^4^/ mL (in 50 mL). A 500 µL suspension amount was used to prepare the dilution for viability plating. The cell suspension was then mixed with equal volumes of TFT stock solution (final concentration of TFT: 3 µg/mL). Then, 200 µL of cell suspension containing TFT were dispensed to each well of 96-well flat-bottom plates for each tested dose and reference compounds. Plating for cloning efficiency/Viability (VP) with non-selective (without TFT) cloning medium was also performed. On 96-well, flat-bottom plates, 200 µL of cell suspension in concentration of 8 cells/mL were plated. All plates were incubated for 12–14 days in standard conditions (5% CO_2_, 37 °C). Following that time, the plates were analyzed. The wells containing viable clones were identified in a Zeiss reversed light microscope and counted. In plates with selective medium (with TFT), the number of wells containing large colonies and the number containing small colonies were scored for the negative and positive controls for BAFASAL^®^ doses.

### 2.6. In Vitro Efficacy Study of Different BAFASAL^®^ Forms in Solid Feed

The in vitro effectiveness of BAFASAL^®^ in reducing *Salmonella* bacteria was tested in a simulated crop environment based on previously published protocols [45]. The BAFASAL^®^ preparation (liquid or powder form) was introduced into the feed by various methods: the liquid form was sprayed on or administered by immersion, while the powder form was mixed directly with the feed. The targeted bacteria was *Salmonella* enterica subsp. enterica serovar Enteritidis 65/S/10 from the collection of Proteon Pharmaceuticals S.A. The feed samples were mash DKA Starter (Mieszanka Paszowa UWM w Olsztynie; Batch: 442/2019) produced by Contractus Pasze Sp. z o.o. Warszawa. Two independent experiments were performed, varying in terms of conditions and suspension solutions applied. In both these experiments, feed samples of 1 g were prepared and divided into 5 groups. Each group consisted of three independent replicates. Three groups were treated with bacteriophages and contaminated with bacteria, one was contaminated with bacteria only (positive control—PC) and one was not treated with phage preparation and was not contaminated (negative control—NC). Feed was immersed in bacteriophage solution (IM), sprayed on with bacteriophage solution (SO), or mixed with bacteriophages in the form of a powder (PM). 

#### 2.6.1. Experiment I

Samples SO and PM were first treated with 0.4 mL or 0.2 g of BAFASAL^®^ respectively, in the amount of 3 × 10^7^ PFU per g of feed, then 1 × 10^3^ CFU (CFU—colony forming units) of *Salmonella* bacteria in 0.25 mL saline solution (0.9% NaCl) were added dropwise per g of feed. The amount of *Salmonella* bacteria was established as to reflect the natural level of feed contamination [46]. Samples were mixed and 0.5 mL buffered peptone water was added, to set the moisture of the sample at 50%. This is because the moisture of feed normally present in chicken crop is between 25–50%. Sample IM was first contaminated with *Salmonella* (1 × 10^3^ CFU of bacteria per g of feed were added dropwise), then 3 × 10^7^ PFU of BAFASAL^®^ per g of feed were added with 0.5 mL buffered peptone water. In the case of PC, feed was contaminated with 1 × 10^3^ CFU of *Salmonella* bacteria per g of feed and mixed with 0.5 mL of buffered peptone water. NC was mixed with 0.5mL of buffered peptone water only. All samples were incubated at room temperature and *Salmonella* levels (CFU/g) were monitored by plating onto XLD agar after 1 h and 6 h of incubation. 

#### 2.6.2. Experiment II

The experimental conditions were modified according to Filho et al. [43,45]. Feed samples were autoclaved before division into experimental groups. Samples SO and PM were first treated with 0.4 mL or 0.2 g of bacteriophage preparation, respectively, in the amount of 3 × 10^7^ PFU per g of feed. Afterwards, 1 × 10^3^ CFU of *Salmonella* bacteria in 0.25 mL saline solution (0.9% NaCl) were added dropwise per g of feed. Samples were mixed and 2.5 mL of saline were added to achieve the appropriate moisture of feed. Sample IM was first contaminated with *Salmonella*, and then 3 × 10^7^ PFU of BAFASAL^®^ per g of feed were added along with saline solution. In the case of PC, feed was contaminated with 1 × 10^3^ CFU of *Salmonella* bacteria per g of feed and mixed with 2.5 mL of saline solution. NC was mixed with saline only. All samples were incubated at 37 °C and bacteria levels (CFU/g) were monitored by seeding on an XLD agar after 1 h and 6 h incubation.

### 2.7. In Vitro Efficacy Study of BAFASAL^®^ in Electrolyte Solution

The study on electrolyte solution experimentally contaminated with *Salmonella* was conducted in conditions reflecting the administration of such liquid via the unit’s drinking water line. The commercial electrolite solution “BIOSTARTER liquid” (matrix, BioPoint, Lot No. 011116), which contains sorbitol, potassium chloride, sodium chloride, mono- and diglycerides of fatty acids, anise oil 20,000 mg/kg, Vitamin C 5000 mg/kg, Vitamin D3 200,000 IU/kg, and betaine 1875 mg/kg was used. The matrix was diluted 2000 times in water (working solution) prior to use, according to the manufacturer’s recommendations. Two experimental groups were tested: T1—positive control (matrix contaminated with *Salmonella*); and T2—experimental group (matrix contaminated with *Salmonella*) treated with BAFASAL^®^ (liquid form). The targeted bacteria were *Salmonella* enterica subsp. enterica serovar Enteritidis 65/S/10 from the collection of Proteon Pharmaceuticals S.A. The study was performed in 50 mL polypropylene centrifuge tubes with 6 replicates per treatment. First, the matrix was diluted and used as working solution for preparing the *Salmonella* and BAFASAL^®^ suspensions. For each replicate of T1 and T2, 10 mL suspensions of *Salmonella* in working solutions of matrix were prepared from the *Salmonella* culture (after the *Salmonella* culture reached OD_600_ = 0.7, it was diluted 200 times giving the concentration 1 × 10^6^ CFU/mL). The same suspension was prepared for the same replicate of each treatment, e.g., for T1A, T2A, etc.

For BAFASAL^®^ suspensions, preparation of 6 individual 10 mL suspensions (50× diluted) in a working solution of matrix were prepared, giving 2 × 10^6^ PFU/mL (2 × 10^7^ PFU/tube). Then, treatment samples were prepared as follows: the 10 mL of matrix working solution was added to 10 mL of each *Salmonella* suspension of T1, while 10 mL of diluted BAFASAL^®^ in the matrix was added to 10 mL of each *Salmonella* suspension of T2. Next, 8 mL from each replicate of T1 and T2 treatments were transferred into fresh tubes for enumeration of *Salmonella spp*. (time 0 h). The remaining volume of samples (12 mL) in each T1 and T2 tubes was incubated in aerobic conditions at 30 °C ± 3 °C for 6 h. After incubation, samples were subjected to *Salmonella spp.* enumeration (time 6 h). *Salmonella spp*. enumeration was performed with the miniaturized Most Probable Number (MPN) technique according to ISO/TS 6579-2.

### 2.8. In Vivo Efficacy Study of Liquid BAFASAL^®^ Preparation

The in vivo efficacy of BAFASAL^®^ was performed on male Ross 308 broilers at the State Scientific-Research Control Institute of Veterinary Preparations and Feed Additives in Lviv, Ukraine. The study was conducted in compliance with the Good Laboratory Practice principles and in accordance with the EFSA guidelines. In this study, 180 one-day-old broilers purchased from a local commercial hatchery were used. Birds were vaccinated against Newcastle Disease at the hatchery and at day 19, against Infectious Bronchitis at the hatchery and at day 10 and against infectious bursal disease at day 13. The study lasted 35 days. Mash feed and water was provided to birds *ad libitum*. There were three groups of birds included in the study: T1—negative control group, T2—positive control group (birds infected with *Salmonella*), T3—treatment group (birds infected with *Salmonella* and receiving BAFASAL^®^ at 2 × 10^6^ PFU/bird/day). All birds belonging to groups T2 and T3 were infected with *Salmonella*. The targeted bacteria were *Salmonella enterica* serovar Enteritidis 12 from the collection of Proteon Pharmaceuticals S.A. It was given on day 4 of the trial directly into crop at a dose of about 5 × 10^4^ CFU/bird, estimated on OD_600_ measurement (OD_600_ = 0.7 diluted 1 × 10^3^ times giving the concentration 2 × 10^5^ CFU/mL). During this trial the following parameters were recorded: pen body weight at 0, 21 and 35 days; average daily weight gain, average daily feed intake, feed conversion ratio (FCR) and pen water intake in the periods 0–21, 22–35 and 0–35 days. Number of *Salmonella* in caeca was assessed at the end of the trial using the most probable number (MPN) technique according to ISO/TS 6579-2. Additionally, daily health records, illness, culls, mortality, including reason for culls and probable cause of mortality, were recorded.

### 2.9. Statistical Analysis

The used statistical tests were adjusted to the conducted studies and analyzed with STATISTICA program (version 13.1, StatSoft, Cracow, Poland).

In order to analyze the differences between the groups in the sub-chronic study, normality tests and analysis of variance were performed. For variables not normally distributed or not having equal variance, the Kruskal–Wallis test was used. In order to identify the group or groups that differ from the others, multiple comparison procedures were applied (Dunn’s method and Holm–Sidak method). For variables that had a normal distribution and equal variance, analysis of the variance test for independent samples (ANOVA for independent groups) was used. 

In the tolerance study, 1-way ANOVA followed by Tukey’s post hoc test, for male and female birds as one group and separately, were used to assess the statistical significance of the results concerning BW (body weight), ADG (average daily gain), ADFI (average daily feed intake), FCR (feed conversion ratio, feed:gain), WI (water intake) and blood parameters. For mortality statistics, the chi-square test was used. 

In the efficacy study, 1-way ANOVA followed by Tukey’s post hoc test was used to assess the statistical significance of the results concerning BW (body weight), ADG (average daily gain), ADFI (average daily feed intake), FCR (feed conversion ratio). For mortality statistics, the chi-square test was used.

Analysis of the MN (micronuclei) frequency and binucleate cells with MN was performed for each treatment using the Chi-square test with Yates’ correction for α = 0.05. In order to examine the dose-response relationship in micronuclei frequencies, the Chi-square test for trends was performed. For cytotoxicity assessment, the cytotoxicity block proliferation index (CBPI) was used.

In the mutagenicity assay all experiments were performed in duplicate: two independent 96-well plates were seeded per condition (test sample/concentration). All results are shown as a mean value. Acceptance criteria were applied and different calculations were carried out according to the OECD guideline 490. The following factors were calculated: RSG (Relative Suspension Growth—an indicator of short-term cytotoxicity), RTG (Relative Total Growth—an indicator of relative cell survival), RS (Relative Survival), and RCE (Relative Cloning Efficiency). These are indicators of cell viability just after treatment and at the time of mutant selection. Moreover, Rate (per cent) of small colonies formed on TFT resistance plates (% small colonies), “fold increase” (as the ratio of mutant frequency for the dose concentration to mutant frequency of the solvent control) and Induced Mutant Frequency were also calculated. The calculation of the above factors was in accordance with the OECD guideline.

For the in vitro efficacy assessment in solid feed, one-way ANOVA followed by Tukey’s post hoc test was applied. In electrolyte solution, Student’s t test was performed in order to assess the statistical significance of received differences in *Salmonella* counts (log10 MPN/mL) before and after BAFASAL^®^ treatment. If the result was 0, it was replaced with half the value of the lowest positive result (lowest positive value was 0.16 MPN/mL, so that negative results were replaced by 0.08 MPN/mL).

In all performed statistical methods, significant differences were declared at *p* ≤ 0.05, while the probability 0.05 < *p* ≤ 0.10 was considered as a near significant trend. 

## 3. Results

### 3.1. BAFASAL^®^ Characterization

**Figure 1:**
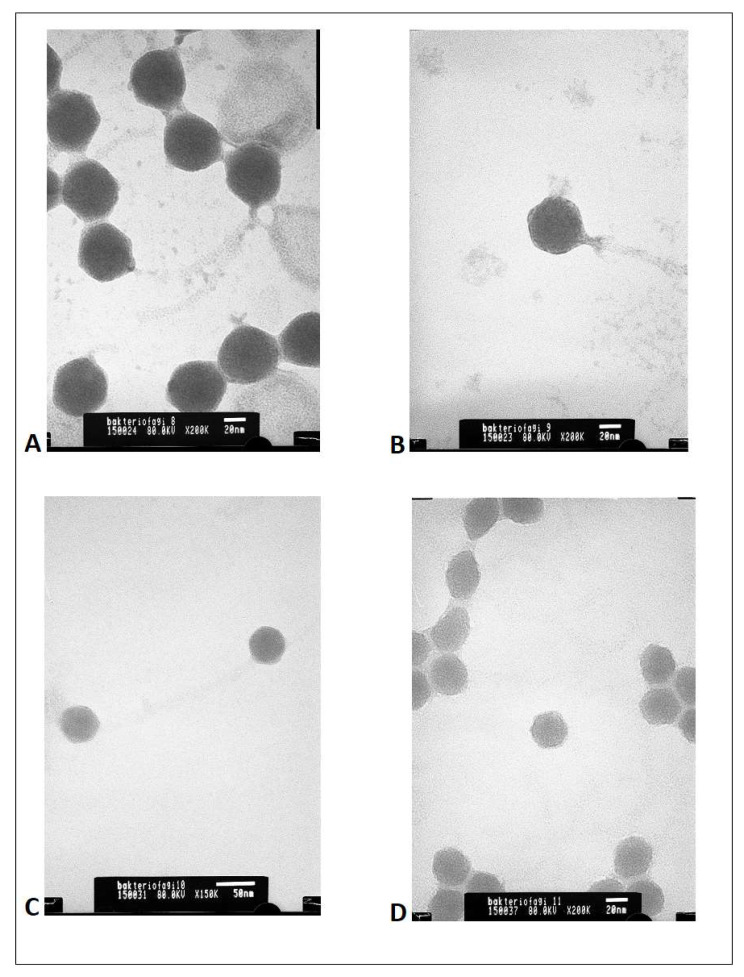
Transmission electron micrograph of (**A**)—3Sent1 (200×); (**B**)—8Sent65 (200×); (**C**)—8Sent1748 (150×); and (**D**)—5Sent1 (200×).

BAFASAL^®^ is a cocktail of four anti-*Salmonella* bacteriophages: 8sent1748, 8sent65, 3sent1, and 5sent1. Each phage included in BAFASAL^®^ was characterized by an EcoRI restriction profile (Supplementary Materials: Figure S1) and TEM micrographs (Figure 1).

Based on the results of these analyses, each phage was proved to be unique, but all four phages, show the morphology of an icosahedral head and a long non-contractile tail. The bioinformatic study on sequences revealed that all bacteriophages contain the genome in the form of dsDNA and belong to *Caudovirales* order. Homologues of all 4 bacteriophage genomic sequences can be found in public international databases and are well described in the literature [47,48,49,50]. Three bacteriophages including 8sent1748, 8sent65 and 3sent1 display high similarity to phages from Genus T5 virus (homologues: *E.coli* virusT5 and *Salmonella* phage vB_SenS_SB6), while 5sent1 displays high similarity to phages from Genus Jerseyvirus (homologue: *Salmonella* phage vB_SenS-Ent1). In the Table 1. the new taxonomic classification of those phages was presented (according to ICTV Taxonomy Release #35: 2019). Moreover, all 4 phages are considered as virulent based on the conducted analysis which revealed no lysogeny modules in their genomes. According to PhageAI, they exhibit lytic lifestyle as well. Their closest reference strains are viruses of Genus T5 (for 3sent1, 8sent65 and 8sent1748) and Jerseyvirus (for 5sent1) with confirmed lytic properties. What is more, all of the studied phages are deprived of any antibiotic resistance or virulence genes according to bioinformatic tools used in this study. Finally, careful analysis of their ORFs excluded transduction potential or any factors coding for toxins.

The liquid preparation contained 1 × 10^8^ of phage particles per mL of product (1 × 10^8^ PFU/mL), while the dry preparation contained 1 × 10^7^ of phage particles per g of product (1 × 10^7^ PFU/g). The analysis of both forms of the preparation showed microbiological sterility of the final products, as well as lack of bacterial production strain genomic residues, which was tested for the presence of genes specific to the production strain. The endotoxins level in final product varied between tested batches from 12 to 435 EU/mL.

Host range was performed on a total of 313 *Salmonella* strains representing 18 serovars using the liquid cocktail preparation (Table 2). The respective host ranges of each individual phage comprising the cocktail was not performed. The preparation showed broad activity against strains of *S.* Enteritidis, *S.* Typhimurium and *S.* Gallinarum. Spot lysis was also observed for a total of 14 of 18 distinct serovars suggesting BAFASAL® is active against a broad range of *Salmonella* serovars. 

### 3.2. Acute Oral Toxicity

The BAFASAL^®^ preparation was administered to female Wistar rats in a single dose of 300 mg/kg b.w. or 2000 mg/kg b.w. In all tested groups, no signs of toxicity were observed. All animals, in each group survived the experiment. Moreover, body weight gain after 14 days of observation was noted in each group. There were no alterations in assessed clinical features, and the gross examinations did not reveal any pathological changes (Table 3). 

### 3.3. Sub-Chronic Oral Toxicity 

The BAFASAL^®^ preparation was administered to male and female Wistar rats at three dose levels—125, 500, 2000 mg/kg b.w. once a day for a period of 90 days. During the entire dosing period, symptoms of illness or toxicity were not observed. As a result of detailed clinical observations, there were no changes in the health status of animals in all examined aspects neither in the study nor the control groups. Moreover, there were no statistically significant differences in body weight between animals treated with BAFASAL^®^, regardless of the dose used, and the control group. Throughout the duration of the experiment, the animals gained weight. In the case of the satellite group, body weight remained at the same level after the end of the preparation administration. There were no differences in response to bacteriophage application between males and females in terms of body weight in comparison to the control group. Other important measurements, i.e., behavioral and neurological parameters, did not reveal any differences among treatment, satellite and control groups. Macroscopic evaluation of the organs from different systems (reproductive, endocrine, nervous, gastrointestinal, etc.) also did not reveal any alterations in terms of structure and general appearance. Furthermore, there were no statistically significant differences in responses of males and females to the examined bacteriophage cocktail at all examined doses, i.e., 125, 500, 2000 mg/kg b.w. in terms of the registered parameters. 

Full histopathological analysis was only performed in animals from the control group and the group receiving BAFASAL^®^ at the dose of 2000 mg/kg b.w. due to lack of pathological changes in all tested organs. Slight histological deviations, however, were noted in some internal organs (kidneys, spleen, thymus, pancreas, small lymph nodes and lungs) of animals receiving the dose of 2000 mg/kg b.w., but only in a few individuals. Those changes were not severe and, according to the histopathologist, they were associated with the termination procedure at the end of the study. Analysis of the material from the satellite group, two weeks after treatment ended, showed a small decrease in the number of leukocyte foci in the lungs, indicating no pathological changes and no activation of the immune system associated with the ongoing inflammation.

Hematological and biochemical analysis revealed only some animal–individual variation in the assessed parameters, which was neither associated with the dose-response effect of BAFASAL^®^ administration nor with pathological state.

There were also no significant deviations in terms of the overall analysis of urine in the test groups of animals in comparison to the control group.

### 3.4. Tolerance Study in Chicken Broilers

This study was performed on male and female Ross 308 chicken broilers. The results showed tolerance of the preparation even in 100× concentrated dose with no statistically significant differences in mortality among experimental groups. The necropsies of birds that died during the trial revealed that the most frequent causes of death were related to the gastrointestinal tract. Similar death rate and causes of death were observed in both the control and BAFASAL^®^-treated groups; thus, the bacteriophage application did not result in increased death rate and did not cause the development of new symptoms. The mortality statistics are presented in Table 4.

Moreover, BAFASAL^®^ administration did not influence water intake. As for other zootechnical parameters, we observed significantly lower body weight and average daily body weight gains in female birds belonging to treatment T4 (application of bacteriophages in the highest tested dose: 2 × 10^8^ PFU/bird/day) after 12 days of trial, in comparison to the control group (T1). This alteration, however, was temporary and revealed primarily that the weight discrepancy effect was no longer observed after 21 days of trial. Furthermore, no dose response effect was demonstrated. We also noticed that during the starter period (0–12 days of trial), the food conversion ratio (FCR) was significantly higher in female birds belonging to T3 and T4 treatments, in comparison to the control group (T1). This difference, however, was not sustained during subsequent periods of the experiment. The FCR in female birds belonging to the T4 treatment, however, tended to be higher in comparison to the T2 treatment, during the Grower II and overall period.

The assessment of blood morphology parameters revealed no significant differences between the examined groups of birds. As for biochemical parameters, in female birds belonging to T2, a higher concentration of aspartate aminotransferase (AST) and alanine transaminase (ALT), in comparison to the control group (T1), was noticed; however, no dose-dependent effect of BAFASAL^®^ on AST and ALT was observed. Uric acid tended to be slightly higher in birds belonging to T2 in comparison to the control group (T1). No other blood biochemistry parameters were influenced by BAFASAL^®^ administration. Detailed information on biochemical and key zootechnical parameters are presented in Table 5.

### 3.5. Genotoxicity Studies Including Mutagenicity 

The combination of two tests has been used to assess the genotoxicity of 50× concentrated BAFASAL^®^: the micronucleus assay (MNA) and the Mouse Lymphoma Assay (MLA). Both genotoxicity tests were preceded by preliminary cytotoxicity tests, which allowed to determine the non-cytotoxic concentration range of BAFASAL^®^. 

During short exposure with metabolic activation, as well as short and extended exposure without metabolic activation, 50× concentrated BAFASAL^®^ did not exhibit cytotoxicity exceeding 55 ± 5%. Thus, in accordance with OECD 487, the following concentrations were chosen for the genotoxicity experiments: 0.25 µL/mL, 0.5 µL/mL, 1 µL/mL, 2 µL/mL and 5 µL/mL. All of the above concentrations were non-cytotoxic.

For the MLA test, the preliminary cytotoxicity assay also revealed the non-cytotoxic effect of our preparation. Based on the results from the range-finding experiments, concentrations of BAFASAL^®^ for the definitive mutagenicity assay were as follows: 0.5 µL/mL, 1.0 µL/mL, 1.5 µL/mL, 2 µL/mL. 

#### 3.5.1. The MNA Test

**Figure 2:**
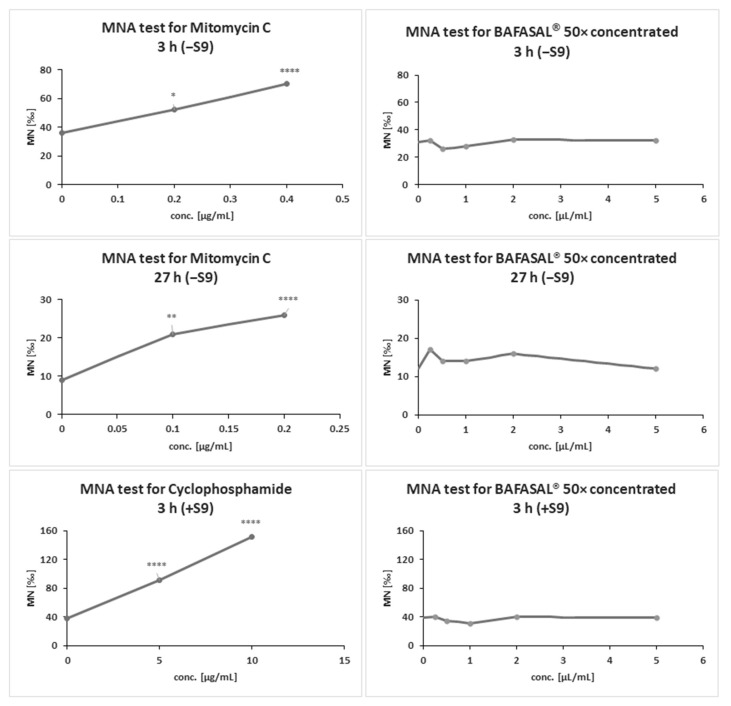
MNA test results for BAFASAL^®^ in concentrated form (50×). CHO-K1 cells were treated with tested items and appropriate negative and positive controls (MMC, CP) in a system with and without metabolic activation, and were scored by microscopy. The asterisk indicates the significant differences in MN frequency compared to concurrent control. Statistically significant level: ns *p* > 0.05; * *p* ≤ 0.05; ** *p* ≤ 0.01; *** *p* ≤ 0.001; **** *p* < 0.0001.

The MNA test results revealed no genotoxicity of 50× concentrated BAFASAL^®^ in any of the experimental conditions tested (Figure 2 and Table 6). Our bacteriophage preparation in a system with and without metabolic activation in tested conditions did not exhibit statistically significant increase in micronucleus frequency per culture compared with the concurrent negative control (*p* > 0.05).

Moreover, no significant concentration-related increase in the frequency of MN was observed in cultures treated with the concentrated BAFASAL^®^ form. MN formation was significantly induced in CHO-K1 cells compared to the control following exposure to positive control (reference items MMC and CP) at indicated concentrations (*p* < 0.05). The number of CHO-K1 cells with MN increased in an MMC/CP exposure concentration-dependent manner (*p* < 0.05). The results obtained from positive control demonstrated the reproducibility and sensitivity of the system used to analyze the genotoxic potential of items.

#### 3.5.2. The MLA Test

**Figure 3:**
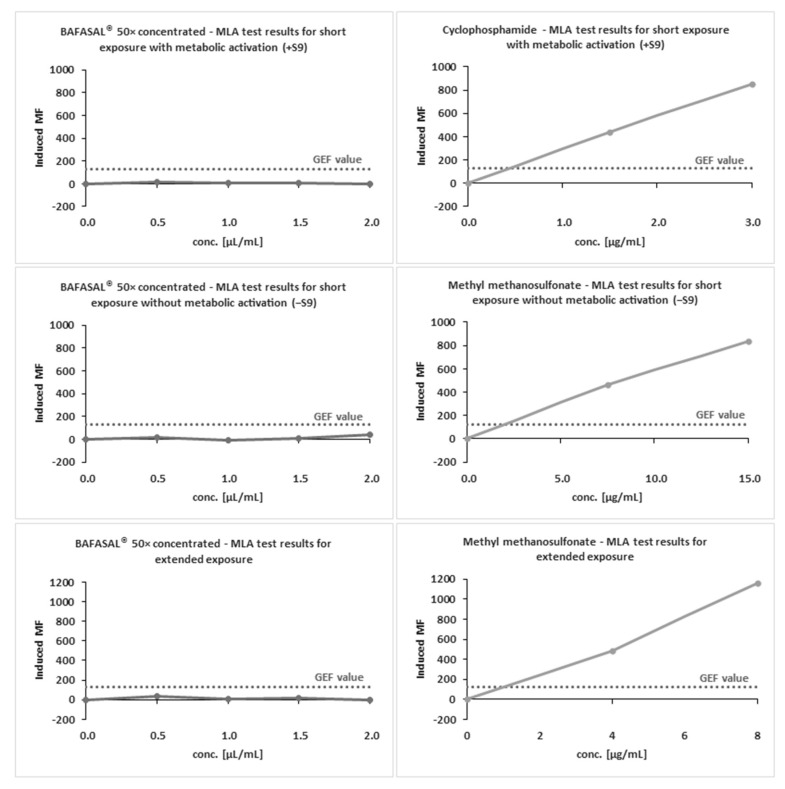
MLA test results for BAFASAL^®^ in concentrated form (50×). L5178Y TK+/− cells were treated with the BAFASAL^®^ preparation, appropriate negative controls and reference items (MMS, CP), in a system with and without metabolic activation and culture in the presence of TFT. Induced MF was calculated on the basis of the number of cell colonies per well after 11–14 days of incubation.

The definitive mutagenicity assay revealed that none of the doses of 50× concentrated BAFASAL^®^ induced dose-related mutagenic effects in mouse lymphoma cells under experimental conditions (Figure 3 and Table 7). In the absence and presence of metabolic activation, the induced mutation frequency (IMF) level did not exceed 126 × 10^−6^ in any of the doses tested. MMS and Cp were used as reference items with and without S9, respectively. Both reference items yielded IMF above 300 × 10^−6^ in TFF-resistant colonies, therefore indicating the assay’s sensitivity and responsiveness to mutagens. 

The obtained results indicate that BAFASAL^®^ (even in 50× concentrated form) is non-mutagenic under the conditions employed and the criteria defined in OECD guidelines (490).

### 3.6. In Vitro Efficacy Assay—Solid Feed

In in vitro efficacy studies, the effectiveness of BAFASAL^®^ in reducing *Salmonella* bacteria was tested in a simulated crop environment. To this end, two different forms (liquid and powder) of BAFASAL^®^ were introduced into the feed, and different ways and conditions for its administration into the feed were tested (spraying on liquid form, immersion in liquid form, or mixing with powder). The number of re-isolated *Salmonella* bacteria in feed samples strongly depended on the form of BAFASAL^®^ used, its way of administration, the time and the temperature of incubation. The general observation, however, is that the application of BAFASAL^®^ in each form resulted in a significant decrease in bacteria numbers per gram of experimentally contaminated feed. 

In experiment I, in which incubation was performed at room temperature (mimicking the normal conditions of feed storage), after one hour of incubation of feed samples with bacteria, we observed a threefold decrease in bacterial numbers in samples, which were first treated with BAFASAL^®^ (BAFASAL^®^ was introduced into the feed by spraying on the liquid form—SO group—or mixing with the powder–PM group) and then inoculated with bacteria. The results are as follows: Δlog10(CFU/g) = 0.49 log and Δlog10 (CFU/g) = 0.46 log for samples sprayed on and mixed with bacteriophage powder respectively, compared to the positive control. Immersed feed samples (IM group), which were first contaminated with *Salmonella* and then treated with BAFASAL^®^, revealed a twofold decrease (Δlog10(CFU/g) = 0.33 log) in *Salmonella* numbers in comparison to the positive control. All results were statistically significant (*p* < 0.05). After 6 h of incubation, the highest efficacy was obtained for the bacteriophage sprayed samples, in which re-isolated *Salmonella* (CFU per g of feed) was more than five times lower (Δlog10 (CFU/g) = 0.72 log) than in the control group. Similar statistically significant results were obtained for samples where BAFASAL^®^ was added post contamination via immersion (IM group): an almost five times lower *Salmonella* number was counted (Δlog10 (CFU/g) = 0.69 log). The powder form of bacteriophage preparation inhibited the multiplication of bacteria fourfold, giving a result close to being statistically significant (*p* = 0.053). 

In experiment II, the conditions were changed to mimic better the internal environment of an organism (incubation at 37 °C). After one hour of sample incubation in higher temperatures, we observed an almost 17 times (1.2 log) lower number of *Salmonella* isolates in the SO group, and an almost 23-fold (Δlog10(CFU/g ) = 1.36 log) decrease in bacteria CFU/g in IM samples, in comparison to the positive control. In PM samples, the decrease was almost 12 times (Δlog10 (CFU/g) = 1.07 log) lower than that of the control. All results were statistically significant (*p* < 0.05). After 6 h of incubation, the inhibitory effect of BAFASAL^®^ persisted. In all phage-treated groups, the decrease in the number of re-isolated *Salmonella* was 10 times lower (almost 1 log in all tested groups) than in the control group. These results were significant with high statistical tendency (*p* = 0.051).

The general observation is that the bacteriophage-based preparation BAFASAL^®^ is efficient in all presented forms in terms of inhibiting *Salmonella* growth in solid feed.

**Figure 4:**
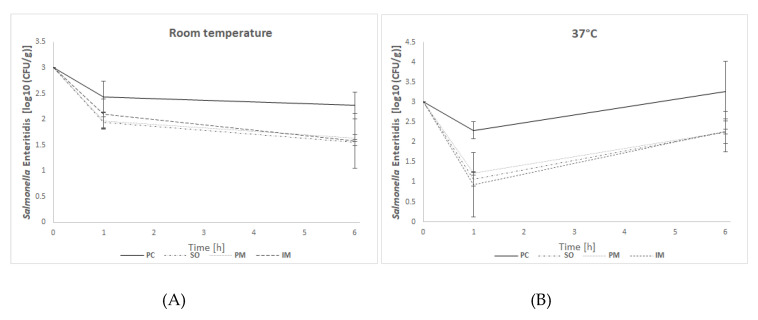
In vitro efficacy results from simulated crop environment (mean values with standard deviations from three independent replicates). PC—Positive control, SO—Feed sprayed on with BAFASAL^®^, PM—Feed mixed with BAFASAL^®^, IM—Feed immersed in BAFASAL^®^. Panel (**A**)—Experiments performed at room temperature; Panel (**B**)—Experiments performed at 37 °C.

Detailed information is presented in Figure 4 and Table 8.

### 3.7. In Vitro Efficacy Assay—Electrolyte Solution

In addition to the in vitro efficacy of BAFASAL^®^ on solid feed, a study on electrolyte solution experimentally contaminated with *Salmonella* was conducted. This study was performed in conditions reflecting the administration of electrolyte solution via the unit’s drinking water line. As a result, we proved that the BAFASAL^®^ preparation was similarly effective as in solid feed. Bacteria were not detected in any of the six replicates after 6 h of incubation, and this result was statistically significant (*p* < 0.0001) (Table 9).

### 3.8. In Vivo Efficacy Study in Chicken Broilers

The results of in vivo efficacy study showed that the number of *Salmonella* in caeca of birds infected with *Salmonella* Enteritidis was significantly reduced (by more than 16 times, Δlog10 (CFU/g) = 1.21 log) in birds receiving BAFASAL^®^ (T3) in comparison to the group not receiving the preparation (T2). Zootechnical parameters were improved after BAFASAL^®^ treatment. Body weight, average daily gain and mean daily feed intake were significantly higher in the group receiving BAFASAL^®^ (T3) than both in the negative control (T1) and in the positive control group (T2), while FCR was significantly lower. The results are presented in Table 10. There was no mortality during the study.

## 4. Discussion

Current global food production and food security are challenged by the increasing presence of antibiotic-resistant bacteria. The transmission of those bacteria to humans and the induction of foodborne diseases pose a serious problem globally, which impacts on both public health and the economy. Some of the estimates predict that in 2050 about 10 million people may die due to infection caused by antimicrobial resistant pathogens [51,52]. Overusing antibiotics, both in human treatment and, even more importantly, in animal husbandry has led to the propagation in the environment of bacteria equipped with sophisticated, versatile microbial defense mechanisms against all relevant antibiotics [53]. This has prompted the scientific community, governments, and public health agencies to prioritize research aimed at the development of novel, alternative ways to control pathogenic bacteria [54]. The application of bacteriophages seems to be one of the leading innovations in this area, garnering increasing interest among medical and food industry experts and the general public [34,54,55,56]. Efforts to reintroduce bacteriophages in order to control bacterial pathogens are being made in human medicine, animal health, food safety, and crop protection [2,3,33,34,56,57,58,59].

One of the requirements for antibacterial preparation to be introduced into the market is their safety confirmation. Even though bacteriophages have been successfully used in human treatment for more than one hundred years [57], changes in regulatory requirements and scientific progress have created a need to develop standards for assessing bacteriophage preparation similarly to other biological products, such as probiotic bacteria and yeast [3,44]. 

In this study, we evaluated the safety and efficacy of the bacteriophage-based preparation BAFASAL^®^, intended to prevent *Salmonella* contamination in food production chain. The preparation showed activity against a broad range of *Salmonella* serovars with broadest activity against strains of ***S.*** Enteritidis, ***S.*** Typhimurium and *S.* Gallinarum which was assessed on end product using spot test methodology. In the future, we plan to perform the host range testing also on individual phages using a more robust method., The safety and efficacy studies were performed in compliance to GLP (Good Laboratory Practice), OECD (Organization for Economic Co-operation and Development) Environment, Health and Safety requirements standards and EFSA (European Food Safety Authority) recommendations. 

Two in vivo toxicity assays, namely oral acute and sub-chronic toxicity evaluations performed on rats, aimed to determine the potential development of bacteriophage-induced toxic effects in living organisms. In the acute toxicity study, where rats were subjected to high, repetitive doses of bacteriophage preparation for 14 days, no signs of acute toxicity were observed. All exposed animals survived the experiment without alteration in clinical features and in gross examination of internal organs in histopathology of analyzed tissues. Similar results were obtained in the sub-chronic oral toxicity study, where no signs of toxic activity of the applied bacteriophages were noted during the entire 90-day experiment. Thus, the bacteriophage preparation tested at the highest dose envisioned in the OECD standard for chemical safety testing, using two different time regimes of exposure is not toxic, suggesting that it is safe for consumers. These data are aligned with available toxicity studies performed with other bacteriophages (single or in cocktail) on different animal species and different times of exposure and observation. Cartlon et al. repeatedly applied a high dosage of bacteriophage P100 to Wistar albino rats. No in-life effects attributable to the material were observed. As with our experiment, no deaths were observed during their experiment, nor any abnormal physical or behavioral signs. Moreover, they also noticed slight histomorphologic deviations within internal organs, just as in our long-term toxicity study experiment, but similarly to our observations, these were assumed to be typical of those, which occur spontaneously in laboratory rats of this strain and age. They concluded that there was no correlation between the appearance of those changes and phage administration [23]. The application of the anti-*Salmonella* bacteriophage wksl3 to BALB/c mice also did not reveal any acute side effects associated with bacteriophage presence in the organism [24]. Similar conclusions regarding the safety of bacteriophages were drawn by Chen et al., who performed acute toxicity assay on BALB/c mice. In this study, animals were intraperitoneally inoculated with PHB02 phage at a dose of 1.0 × 10^8^ PFU, and observed for seven days without any abnormalities in their behavior noted [26]. Mice experimental groups for toxicity assessment were also used in the safety evaluation of a mix of three specific *E. coli* bacteriophages (administered via gastric perfusion) and again, after three weeks of observation, no toxic signs of phage activity were revealed. It was concluded that the phage cocktail may be used as a safe preparation for pathogenic *E. coli* bacteria control, which is also in accordance with our observations for our phage cocktail specific to *Salmonella* [25]. Furthermore, the administration of other phage preparations, for example the specific bovine-associated Staphylococcus aureus phage cocktail administered to healthy mice also appeared to be safe and well tolerated [60]. Other research groups, which performed long-term toxicity studies in mice using phage cocktails against different types of bacteria, also confirmed the general assumption that phage administration has no side effects on animal health, their life parameters, and behavior [61,62,63,64,65]. Rodents are used in toxicity studies as standard; therefore, such studies are the most valuable from the point of view of risk assessment. The potential adverse effects of bacteriophages, however, were also investigated in other animal species. Drilling et al. examined this aspect for a phage cocktail against ***S. aureus*** in healthy sheep and just like others, did not notice any change in animal general wellbeing. Moreover, as phages were applied to the frontal sinus region, there was no change in the architecture of the sinus mucosal lining, while the profile of immune cells in the sinus mucosa was not increased or altered [66,67]. Furthermore, Soffer et al. examined the anti-*Salmonella* phages on pets (dogs and cats), and did not find any adverse effects in animals after phage consumption via pet food [37]. In our study, we have also performed a tolerance assay on chicken broilers investigating the potential effects of increasing the dosage of the BAFASAL^®^ preparation applied to target animals. The doses were 10× and 100× higher than doses employed in efficacy assays in solid feed and electrolyte solution. Based on the results, we have concluded that the administration of phages was well tolerated at all examined doses, without any adverse effects that could be correlated with the bacteriophages’ treatment. These findings are aligned with other scientific reports determining the safety of bacteriophage application in chicken broilers [25,68,69,70,71]. For the final safety assessment of BAFASAL^®^, we conducted two genotoxicity studies, i.e., the micronucleus test (MNA), and the Mouse Lymphoma Assay (MLA). To the best of our knowledge, the present studies are the first evaluation of the genotoxic potential of bacteriophage preparation. Both studies were performed using internationally validated test methods in accordance with European legislation, OECD Guidelines (OECD 490 and OECD 487), and the principles of GLP. In the opinion of the EFSA Scientific Committee, the combination of these two tests fulfils three genetic endpoints: the MLA covers gene mutations and the MNA assay covers both structural and numerical chromosome aberrations [72]. What is more, these tests are reliable for the detection of the most common genotoxic substances. As both tests showed no limiting cytotoxicity, the study was conducted using the highest recommended concentration of 2 μL/mL, which corresponds to OECD Guidelines. Moreover, the concentration of 5 μL/mL was also tested in MLA to cover a broader range of BAFASAL^®^ concentrations. Our results in the MNA assay revealed that even 50× concentrated BAFASAL^®^ did not induce concentration-dependent genetic toxicity in CHO-K1 cells. Similarly, neither BAFASAL® nor its metabolic derivatives showed genotoxicity when evaluated by the MLA test. Therefore, our results strongly confirm the general assumption that bacteriophages are safe and can be an attractive solution to combating pathogenic bacteria in light of increasing antibiotic resistance.

It is worth emphasizing that the general safety of phage administration to animals can also be supported by the absence of deterioration of the animal state during bacterial infection followed by phage treatment. There are numerous publications studying the efficacy of phage treatment, none of which have indicated any side effects or increase in mortality after the application of phage-based preparations to animals [73,74,75,76].

Further evidence of phage safety can be found in multiple clinical reports. Compassionate use of bacteriophage therapy in patients suffering from antibiotic-resistant bacterial infections in clinics in Tibilisi, Georgia and Wrocław, Poland provides data from the therapeutic application of bacteriophages in several thousands of cases without any significant undesirable side effects [54,76,77,78]. The excellent safety profile of bacteriophages is confirmed by more recent data from other medical centers [28,29,32,79]. Taken together, multiple human clinical studies, including on healthy volunteers, have confirmed the high level of safety of bacteriophage treatment in humans [28,32,80].

Another aspect of using bacteriophage preparations as an antimicrobial solution in different clinical and non-clinical applications is their effectiveness in controlling pathogenic bacteria. In the current study, the efficacy of BAFASAL^®^ applied in different forms (liquid, powder, spray) in different feed types (solid and supplemental liquid) was investigated. Data obtained in experiments reflecting field conditions of storing and using poultry feed indicated a significant decrease in the number of viable *Salmonella* bacilli following artificial contamination of feed with *Salmonella* Enteritidis. The efficacy of BAFASAL® in decreasing the number of *Salmonella* was noticeable within a short period and in all examined conditions. Moreover, this effect was sustained and even enhanced in the following hours, especially while incubating at RT. We also noticed that administration of BAFASAL® before experimental feed contamination, gave better results in terms of *Salmonella* reduction compared to the treatment of already contaminated feed samples. These observations indicate that BAFASAL^®^ can be used as a feed protective agent (in prophylaxis against *Salmonella* feed contamination) affecting food safety. In the case of electrolyte solution, the addition of this preparation resulted in the complete elimination of *Salmonella* within 6 h. This is similar to the previously reported effects of other bacteriophage preparations employed to protect broiler feed from *Salmonella* Enteritidis contamination, where complete elimination of bacteria was observed [45]. There are also reports of the effective prevention of *Salmonella* contamination by bacteriophage preparations in other types of feed, such as dry pet feed [36]. It is worth mentioning that a significant effect of bacteriophages applied in pet food was detected using similar doses of anti-*Salmonella* bacteriophages [36] as those applied in the current study. Moreover, consistent with our observations, the authors did not notice any visible changes in terms of feed appearance, smell and structure. Similar observations regarding the efficacy and non-disturbance of the food’s visual appearance were reported by Soffer et al. in a variety of raw pet food (turkey meat, chicken, lettuce, tuna) [37]. Thus, the in vitro efficacy assay revealed that the BAFASAL^®^ preparation is effective in decreasing the number of live *Salmonella* bacilli in poultry feed, which is aligned with previously reported data on different bacteriophage preparations tested in poultry and other animal feeds.

Moreover, the results of in vivo efficacy study showed that treatment with BAFASAL^®^ significantly decreased the *Salmonella* content in caeca of birds infected with *Salmonella* Enteritidis in comparison to the group not receiving the preparation. Similarly, Kim et al. demonstrated that anti-*Salmonella* Enteritidis bacteriophage supplemented in basal diet (0.05, 0.1 and 0.2% of total diet) decreased *Salmonella* Enteritidis concentration in the cecum [70]. Seo et al. proved that the application of *Salmonella*-specific bacteriophage cocktail efficiently controls the *Salmonella* levels in pigs and does not have any significant effect on their microbiome [73]. In similar studies, Bardina et al. showed the effectiveness of a bacteriophage cocktail in reducing the concentration of *S*. Typhimurium in the chicken cecum [81]. Moreover, Borie et al. reported a significant reduction in *Salmonella* Enteritidis incidence in experimentally infected chickens treated with a combination of three different bacteriophages in drinking water [82]. The effectiveness of bacteriophage treatments was also confirmed by Nabil et al., who demonstrated the decrease in *Salmonella* colonization in the cecum of infected chickens after administration of five phage doses [83]. 

Detailed examination of BAFASAL^®^ confirmed the general assumption of bacteriophages as harmless to animals and effective in the struggle against bacteria, which pose a public health concern, and proved that BAFASAL^®^ can be used as feed protective agent affecting food safety.

## 5. Conclusions

Bacteriophages are believed to play a key role in establishing microbial balance in every ecosystem by controlling the number of bacteria. They are considered a viable alternative to antibiotics and other chemical antimicrobials used in different setups. Data obtained from extensive testing of the safety and efficacy of BAFASAL^®^—a bacteriophage-based anti-*Salmonella* preparation revealed that it possesses desirable characteristics in terms of safety and efficacy requirements. The outcomes of acute and sub-chronic oral toxicity, genotoxicity and tolerance testing in targeted species confirmed experimentally the high level of safety of this particular bacteriophage preparation for consumers and animals, which is consistent with the general assumption that bacteriophages are inherently safe for humans and animals based on their biology, long history of clinical applications, and multiple experimental studies. At the same time, the application of the BAFASAL^®^ bacteriophage cocktail in solid and fluid poultry feed has the potential to reduce *Salmonella* feed contamination effectively, thus affecting the safety of the food production chain. What is more, the in vivo usage of the preparation is highly effective in decreasing the *Salmonella* content in caeca of birds infected with *Salmonella*.

## Figures and Tables

**Table 1:** Genomic features of bacteriophages.

Feature	8sent1748	8sent65	3sent1	5sent1
Taxonomy	Caudovirales; Demerecviridae; Markadamsvirinae; Tequintavirus	Caudovirales; Demerecviridae; Markadamsvirinae; Tequintavirus; unclassified Tequintavirus	Caudovirales; Demerecviridae; Markadamsvirinae; Tequintavirus	Caudovirales; Siphoviridae; Guernseyvirinae; Jerseyvirus
Genome size (bp)	110,720	112,133	109,746	43,760
Predicted lifestyle	lytic	lytic	lytic	lytic
No. predicted genes () ^a^ () ^b^	161 (49) ^a^ (112) ^b^	164 (41) ^a^ (123) ^b^	156 (50) ^a^ (116) ^b^	60 (40) ^a^ (20) ^b^
Coding region (%)	86.75	86.62	86.61	91.76
G + C content (%)	39.29	39.97	38.96	49.90
Accession number	MT653146	MT653145	MT653143	MT653144

Notes: ^a^—Plus strand, ^b^—Minus strand.

**Table 2:** BAFASAL^®^ spectrum of specificity.

*Salmonella Enterica Serovars*	No of Tested Isolates	No of Sensitive Isolates	% of Sensitive Isolates
Enteritidis	128	126	98
Typhimurium	103	99	96
Agona	2	2	100
Alachua	1	0	0
Brandenburg	1	1	100
Hadar	3	3	100
Heidelberg	2	2	100
Infantis	9	4	57
Kentucky	2	2	100
Molade	1	0	0
Norwich	1	0	0
Paratyphi	1	0	0
Reading	1	1	100
Schwarzengrund	1	1	100
Senftenberg	1	1	100
St. Paul	1	1	100
Virchow	8	5	63
Gallinarum	47	38	80

**Table 3:** Mortality and clinical observations in acute oral toxicity study.

Acute Oral Toxicity			Clinical Signs							
			Locomotor System, Behavior, Reactions to Stimuli	Skin and Hair	Eyes and Eyelids	Respiratory System	Digestive System	Urinary System	Reproductive System	Mortality
Stage	Dose	No of Animals								
I	300	3	NC	NC	NC	NC	NC	NC	NC	0
II	300	3	NC	NC	NC	NC	NC	NC	NC	0
III	2000	3	NC	NC	NC	NC	NC	NC	NC	0
IV	2000	3	NC	NC	NC	NC	NC	NC	NC	0

Notes: NC—No change.

**Table 4:** Mortality statistics (0–35 days) in tolerance study.

Group	Mortality (%)
T1	3.125
T2	4.915
T3	4.330
T4	4.375
*p* value	0.555 (NS)
T1 male	3.750
T2 male	7.955
T3 male	6.250
T4 male	6.875
*p* value	0.629 (NS)
T1 female	2.500
T2 female	1.875
T3 female	2.411
T4 female	1.875
*p* value	0.450 (NS)

Notes: 16 pens (8 male and 8 female) of 20-broilers per treatment; NS—Not statistically significant.

**Table 5:** Biochemical and zootechnical parameters registered during tolerance study.

Treatment	Blood Biochemistry		Feed Conversion Ratio (FCR) in Rearing Periods	
	Aspartate Amino Transferase, U/L	Alanine Amino Transferase, U/L	0–12 days [kg/kg]	0–35 days [kg/kg]
T1 males + females	485.5 ± 138.3	25.5 ± 5.0	1.117 ^b^ ± 0.023	1.500 ± 0.033
T2 males + females	606.1 ± 169.5	26.9 ± 5.1	1.112 ^b^ ± 0.019	1.493 ± 0.049
T3 males + females	590.3 ± 133.5	26.0 ± 4.4	1.142 ^a^ ± 0.027	1.521 ± 0.036
T4 males + females	540.8 ± 157.1	26.8 ± 3.1	1.133 ^a,b^ ± 0.027	1.526 ± 0.093
*p* value	0.125 (NS)	0.800 (NS)	0.003	0.313 (NS)
T1 male	508.6 ± 140.3	27.5 ± 6.1	1.113 ± 0.021	1.476 ± 0.029
T2 male	470.4 ± 107.7	24.5 ± 5.3	1.106 ± 0.025	1.470 ± 0.060
T3 male	563.5 ± 102.8	25.5 ± 5.1	1.132 ± 0.033	1.508 ± 0.045
T4 male	532.0 ± 208.9	27.1 ± 4.2	1.113 ± 0.017	1.472 ± 0.044
*p* value	0.635 (NS)	0.630 (NS)	0.203 (NS)	0.321 (NS)
T1 female	459.1 ^b^ ± 141.8	23.5 ^b^ ± 2.9	1.122 ^b^ ± 0.026	1.524 ^x,y^ ± 0.016
T2 female	761.3 ^a^ ± 29.2	29.3 ^a^ ± 3.7	1.119 ^b^ ± 0.008	1.517 ^y^ ± 0.020
T3 female	617.1 ^a,b^ ± 161.2	26.5 ^a,b^ ± 4.0	1.153 ^a^ ± 0.016	1.535 ^x,y^ ± 0.020
T4 female	549.6 ^b^ ± 95.3	26.4 ^a,b^ ± 1.8	1.153 ^a^ ± 0.017	1.579 ^x^ ± 0.099
*p* value	<0.001	0.014	<0.001	0.097

Notes: Samples collected from 1 bird per pen (16 samples per treatment). Different superscripts denote statistically significant differences or trends (a/b: *p* < 0.05; x/y: 0.05 ≤ *p* < 0.1). T1—Control diet; T2—Control + BAFASAL^®^ at 2 × 10^6^ PFU/bird/day; T3—Control + BAFASAL^®^ at 2 × 10^7^ PFU/bird/day; T4—Control + BAFASAL^®^ at 2 × 10^8^ PFU/bird/day.

**Table 6:** MNA test results for 50× concentrated BAFASAL^®^.

Test Item	CBPI	RI [%]	Cytotoxicity%	MN [‰]	*p* Value	Cells with MN [‰]	*p* Value	Result
3 h (−S9)								
PBS control	1.97	100	0	36	NA	32	NA	NA
0.2 µg/mL MMC	1.89	92	8	52	0.0243 (*)	45	0.0366 (*)	positive
0.4 µg/mL MMC	1.91	93.7	6.3	70	<0.0001 (****)	64	0.0001 (****)	positive
H_2_O control	1.95	100	0	31	NA	28	NA	NA
0.25 µL/mL BAFASAL^®^ 50×	1.97	102.2	−2.2	32	0.9279	31	0.9243	negative
0.5 µL/mL BAFASAL^®^ 50×	1.98	103.8	−3.8	26	0.9448	25	0.7269	negative
1 µL/mL BAFASAL^®^ 50×	1.99	104.5	−4.5	28	0.3837	24	0.6028	negative
2 µL/mL BAFASAL^®^ 50×	2.03	109.3	−9.3	33	0.6002	30	0.5204	negative
5 µL/mL BAFASAL^®^ 50×	2.06	11.9	−11.9	32	0.7561	29	0.8099	negative
27 h (−S9)								
PBS control	1.83	100	0	9	NA	9	NA	NA
0.1 µg/mL MMC	1.78	94.9	5.1	21	0.0033 (**)	20	0.0039 (***)	positive
0.2 µg/mL MMC	1.83	100.8	−0.8	26	<0.0001 (****)	24	0.0002 (***)	positive
H_2_O control	1.82	100	0	12	NA	12	NA	NA
0.25 µL/mL BAFASAL^®^ 50×	1.85	104.4	−4.4	17	0.8846	15	0.8846	negative
0.5 µL/mL BAFASAL^®^ 50×	1.85	104.7	−4.7	14	0.2936	13	0.5003	negative
1 µL/mL BAFASAL^®^ 50×	1.79	96.4	3.6	14	0.6833	13	0.8924	negative
2 µL/mL BAFASAL^®^ 50×	1.84	102.9	−2.9	16	0.6807	14	0.8896	negative
5 µL/mL BAFASAL^®^ 50×	1.87	106	−6	12	0.3558	11	0.5889	negative
3 h (+S9)								
PBS control	1.86	100	0	38	NA	35	NA	NA
5 µg/mL CP	1.74	86.7	13.3	91	<0.0001 (****)	86	<0.0001 (****)	positive
10 µg/mL CP	1.64	74.6	25.4	152	<0.0001 (****)	143	<0.0001 (****)	positive
H_2_O control	1.84	100	0	39	NA	35	NA	NA
0.25 µL/mL BAFASAL^®^ 50×	1.84	99.7	0.3	40	0.9354	37	0.932	negative
0.5 µL/mL BAFASAL^®^ 50×	1.85	101	−1	35	0.8947	32	0.7669	negative
1 µL/mL BAFASAL^®^ 50×	1.92	109.2	−9.2	31	0.5972	29	0.7181	negative
2 µL/mL BAFASAL^®^ 50×	1.84	99.5	0.5	40	0.259	39	0.3324	negative
5 µL/mL BAFASAL^®^ 50×	1.92	110.1	−10.1	39	0.8891	36	0.5846	negative

Notes: Statistically significant levels: ns *p* > 0.05; * *p* ≤ 0.05; ** *p* ≤ 0.01; *** *p* ≤ 0.001; **** *p* < 0.0001.

**Table 7:** The MLA test results for 50× concentrated BAFASAL^®^.

MLA (+S9) 4 h										
Test Item	Conc	RSG	RTG	RCE	RS	Colony Counts	% Small Colonies	MF [1 × 10^−6^]	Fold Increase	IMF [1 × 10^−6^]
BAFASAL^®^ 50×	2.0 µL/mL	104%	105%	95%	102%	20	25%	56	0.95	−3
	1.5 µL/mL	88%	95%	89%	108%	22	41%	66	1.12	7
	1.0 µL/mL	89%	95%	98%	106%	25	44%	69	1.17	10
	0.5 µL/mL	99%	104%	91%	105%	25	40%	71	1.21	12
	0.0 µL/mL ^#^	100%	100%	100%	100%	23	26%	59	1	0
CP	3.0 µg/mL	35%	12%	47%	35%	115	70%	909	15.19	849
CP	1.5 µg/mL	70%	35%	89%	51%	108	60%	503	8.4	443
CP	0.0 µg/mL ^^^	100%	100%	100%	100%	18	50%	60	1	0
MLA (−S9) 4 h										
BAFASAL^®^ 50×	2.0 µL/mL	110%	95%	83%	86%	29	38%	103	1.69	42
	1.5 µL/mL	107%	93%	98%	86%	24	29%	71	1.17	10
	1.0 µL/mL	119%	104%	98%	88%	19	37%	53	0.86	−8
	0.5 µL/mL	107%	103%	87%	97%	21	38%	73	1.2	12
	0.0 µL/mL ^#^	100%	100%	100%	100%	21	57%	61	1	0
MMS	15.0 µg/mL	65%	46%	56%	71%	181	72%	896	14.39	834
MMS	7.5 µg/mL	73%	87%	90%	120%	168	70%	527	8.47	465
MMS	0.0 µg/mL ^^^	100%	100%	100%	100%	25	32%	62	1	0
MLA (−S9) 24 h										
BAFASAL^®^ 50×	2.0 µL/mL	107%	102%	110%	95%	27	37%	71	0.95	−4
	1.5 µL/mL	106%	104%	97%	98%	28	36%	87	1.17	12
	1.0 µL/mL	92%	89%	105%	97%	27	41%	80	1.08	6
	0.5 µL/mL	94%	97%	93%	103%	33	36%	112	1.51	38
	0.0 µL/mL ^#^	100%	100%	100%	100%	25	36%	75	1	0
MMS	7.5 µg/mL	37%	11%	41%	30%	130	59%	1248	14.93	1164
MMS	3.75 µg/mL	76%	47%	77%	63%	122	73%	568	6.8	485
MMS	0.0 µg/mL ^^^	100%	100%	100%	100%	27	41%	84	1	0

Notes: ^#^ WFI water 1% (*v*/*v*); ^^^ PBS 1% (*v*/*v*).

**Table 8:** Results from in vitro efficacy assay in solid feed.

Experiment I (Incubation at RT)		PC	BAFASAL^®^ Concentration 3 × 10^7^ PFU/g		
*Salmonella* Enteritidis initial dose [log10 (CFU/g)]	Incubation time (h)		SO	PM	IM
3.00	1	2.44	1.94 *	1.97 *	2.1 *
	6	2.27	1.55 *	1.63 ^^^	1.58 *
Experiment II (incubation at 37 °C)		PC	BAFASAL^®^ concentration 3 ×10^7^ PFU/g		
*Salmonella* Enteritidis initial dose [log10 (CFU/g)]	Incubation time (h)		SO	PM	IM
3.00	1	2.29	1.07 *	1.22 *	0.93 *
	6	3.27	2.26 ^^^	2.26 ^^^	2.27 ^^^

Notes: *—Statistical significance in comparison to PC; *p* < 0.05; ^^^—High statistical tendency *p* = 0.052. Values presented are base 10-logarithm of *Salmonella* recovered; PC—Positive control, SO—Liquid sprayed on the feed, PM—Feed mixed with the powder, IM—Feed immersed in liquid.

**Table 9:** Results from in vitro efficacy assay in liquid complementary feed.

Parameter	T1—Control (*Salmonella* Enteritidis)	T2–*Salmonella* Enteritidis + BAFASAL^®^	*p*-Value
log10 (MPN/mL) at time 0 h	5.87	6.02	0.3448
log10 (MPN/mL) at time 6 h	7.27	0.18	<0.0001
Δlog10 (MPN/mL) time 0 to 6 h	1.4	−5.84	<0.0001

Notes: MPN—Most probable number.

**Table 10:** Results of in vivo efficacy study.

Group	T1 (Negative Control)	T2 (Positive Control)	T3 (BAFASAL^®^-Treated Group)	*p* Value
Body weight, day 35, g	1,77 ^a^ ± 57	1720 ^a^ ± 66	1925 ^b^ ± 31	<0.0001
Average daily gain, 0–35 days, g/bird/day	49.60 ^a^ ± 1.64	48.12 ^a^ ± 1.89	53.97 ^b^ ± 0.90	<0.0001
Mean daily feed intake, 0–35 days, g/bird/day	70.4 ^a^ ± 1.1	70.3 ^a^ ± 4.2	78.1 ^b^ ± 1.4	<0.0001
FCR, kg/kg	1.95 ^a^ ± 0.08	2.03 ^a^ ± 0.07	1.75 ^b^ ± 0.03	<0.0001
*Salmonella* number in caeca, Log_10_ (MPN/g)	Not detected	1.69 ^a^ ± 0.86	0.48 ^b^ ± 0.85	0.0337

Notes: ^a,b^ Different superscripts in the same row denote statistically significant differences.

## Supplementary Materials

The following are available online at https://www.mdpi.com/1999-4915/12/7/742/s1, Figure S1: Restriction profiles after EcoRI digestion; (A) order: Ladder 1kb, 3Sent1, 8Sent1748, 8Sent65, 5Sent1; (B) order: Ladder 1kb, 3Sent1, 8Sent65, 8Sent1748; ladder 1kb contains following weights from the top: 10 kb; 8 kb; 6 kb; 5 kb; 4 kb; 3.5 kb; 3 kb; 2.5 kb; 2 kb; 1.5 kb; 1 kb; 750 bp; 500 bp; and 250 bp.
