# Supplementary material for: Comprehensive Evaluation of the Safety and Efficacy of BAFASAL® Bacteriophage Preparation for the Reduction of Salmonella in the Food Chain
Source: Viruses. 2020 Jul 10;12(7):742. doi: 10.3390/v12070742 (PMC7412135; doi:10.3390/v12070742)
Supplement: Supplementary file 1 [file viruses-12-00742-s001.pdf]

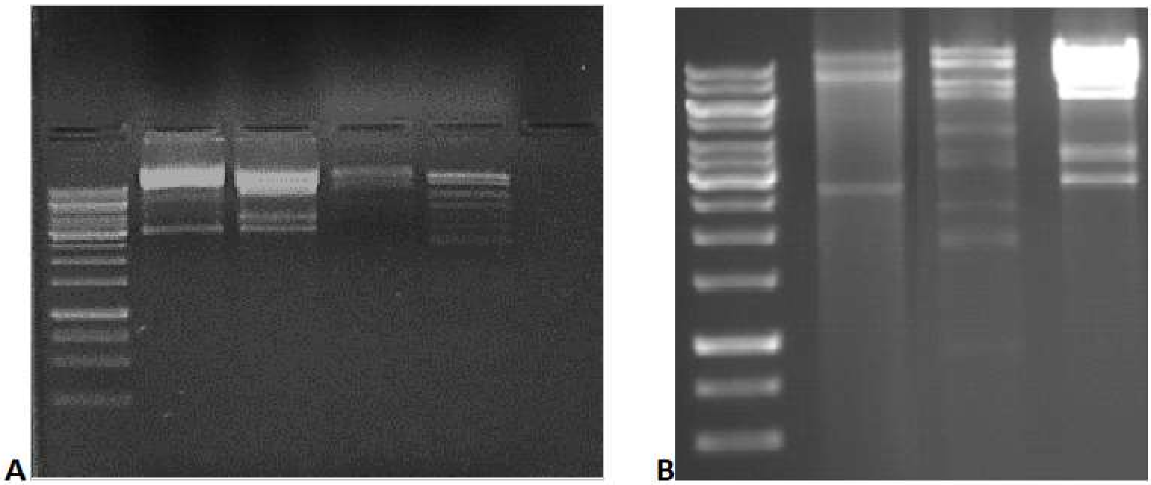

Figure S1: Restriction profiles after EcoRI digestion; (**A**) order: Ladder 1kb, 3Sent1, 8Sent1748, 8Sent65, 5Sent1; (**B**) order: Ladder 1kb, 3Sent1, 8Sent65, 8Sent1748; ladder 1kb contains following weights from the top: 10 kb; 8 kb; 6 kb; 5 kb; 4 kb; 3.5 kb; 3 kb; 2.5 kb; 2 kb; 1.5 kb; 1 kb; 750 bp; 500 bp; and 250 bp.
